# Animal Toxins: A Historical Outlook at the Institut Pasteur of Paris

**DOI:** 10.3390/toxins15070462

**Published:** 2023-07-19

**Authors:** Michel R. Popoff, Grazyna Faure, Sandra Legout, Daniel Ladant

**Affiliations:** 1 Unité des Toxines Bactériennes, Institut Pasteur, Université Paris Cité, CNRS UMR 2001 INSERM U1306, F-75015 Paris, France; 2 Unité Récepteurs-Canaux, Institut Pasteur, Université Paris Cité, CNRS UMR 3571, F-75015 Paris, France; fgrazyna@pasteur.fr; 3 Centre de Ressources et Information Scientifique, Institut Pasteur, Université Paris Cité, F-75015 Paris, France; sandra.legout@pasteur.fr; 4 Unité de Biochimie des Interactions Macromoléculaires, Institut Pasteur, Université Paris Cité, CNRS UMR 3528, F-75015 Paris, France; daniel.ladant@pasteur.fr

**Keywords:** Pasteur, animal toxin, venom, vaccine, serotherapy, therapeutic peptides, phospholipase A_2_

## Abstract

Humans have faced poisonous animals since the most ancient times. It is recognized that certain animals, like specific plants, produce toxic substances that can be lethal, but that can also have therapeutic or psychoactive effects. The use of the term “venom”, which initially designated a poison, remedy, or magic drug, is now confined to animal poisons delivered by biting. Following Louis Pasteur’s work on pathogenic microorganisms, it was hypothesized that venoms could be related to bacterial toxins and that the process of pathogenicity attenuation could be applied to venoms for the prevention and treatment of envenomation. Cesaire Phisalix and Gabriel Bertrand from the National Museum of Natural History as well as Albert Calmette from the Institut Pasteur in Paris were pioneers in the development of antivenomous serotherapy. Gaston Ramon refined the process of venom attenuation for the immunization of horses using a formalin treatment method that was successful for diphtheria and tetanus toxins. This paved the way for the production of antivenomous sera at the Institut Pasteur, as well as for research on venom constituents and the characterization of their biological activities. The specific activities of certain venom components, such as those involved in blood coagulation or the regulation of chloride ion channels, raises the possibility of developing novel therapeutic drugs that could serve as anticoagulants or as a treatment for cystic fibrosis, for example. Scientists of the Institut Pasteur of Paris have significantly contributed to the study of snake venoms, a topic that is reported in this review.

## 1. Introduction

Toxins and poisonings have been known since the most ancient times. During the Paleolithic era in Europe, hunters used poisons. Indeed, paleontologists have discovered unusual grooves on the tips of certain bones used as arrows, suggesting they contained plant or animal poisons. The use of poisons, predominantly from a plant origin, on arrowheads appears to have been widespread among various civilizations across all continents for hunting purposes [1]. The term “toxin” is derived from the Greek word “toxon”, which refers to a bow, implying poisoned arrows, while “poison” is a broader term originating from the Latin “potionem” (meaning a drink). Initially, it denoted harmful liquid substances, and later, it encompassed any dangerous substances. The earliest civilizations were familiar with animal poisons, often using them in combination with plant toxins for hunting and fishing. They also recognized toxins for their potential therapeutic or psychoactive effects, capable of inciting fury, trances, love, and ecstasy. Ancient medical texts such as the Indian Vedic books from 1500 BC demonstrate a considerable knowledge of poisonous substances, including animal poisons, during this era [1]. The term “venom” originates from the Latin name Venus, the goddess of love and beauty. Initially, the word was used to indicate a love potion. Later, it adopted a more ambiguous meaning as a remedy, psychoactive drug, or poison [2]. Nowadays, venom refers specifically to animal poisons delivered through biting. There is a remarkable diversity of venomous animals, including approximately 1450 species of fish, 1200 species of marine organisms, 400 snakes, 200 spiders, 75 scorpions, and 60 ticks [1]. This review is focused on the contribution made by some scientists of the Institut Pasteur of Paris to our understanding of animal toxins.

## 2. Pasteur’s Era: The Discovery of Antivenom Serotherapy

Louis Pasteur was aware of the work on bacterial toxins conducted by Peter Ludvig Panum and von Bergman, who focused on toxins produced by putrefying bacteria [3]. However, L. Pasteur was primarily interested in preventing infectious diseases with attenuated microorganisms. For L. Pasteur, the main mechanism of pathogenicity was the development of microbes in the host, causing a depletion of vital substances [3].

**Figure 1:**
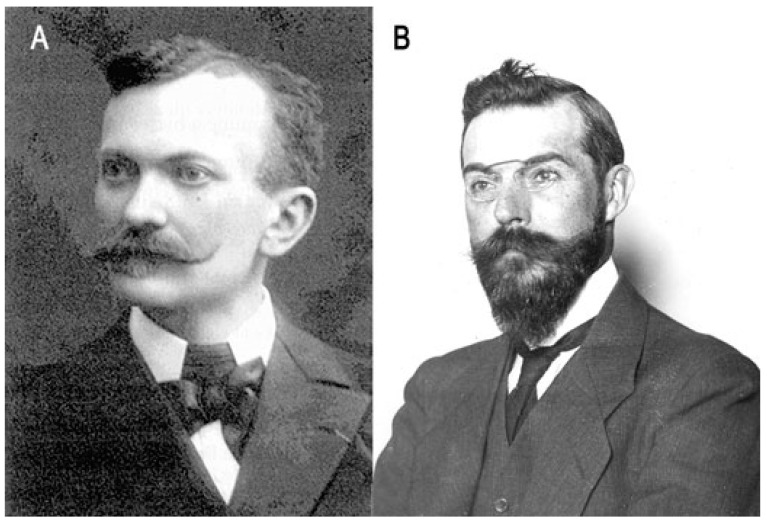
Cesaire Phisalix and Gabriel Bertrand. (**A**) C. Phisalix (1852–1906); photo was taken by Marie Phisalix, Musée d’Histoire Naturelle de Moutiers–Haute–Pierre. (**B**) G. Bertrand (1867–1962) around 1905; photo was provided by the Institut Pasteur/Musée Pasteur.

In 1888, Cesaire Phisalix (1852–1906), a French military physician and scientist, joined the National Museum of Natural History (MNHN, Museum National d’Histoire Naturelle) in Paris in the laboratory of comparative pathology directed by Auguste Chauveau. The MNHN was created in 1636 as the royal garden for the preservation and training of medicinal plants. It was formally established as the National Museum of Natural History in 1793 during the French Revolution. Its activity extends to research, collection, and training in various fields such as botany, chemistry, mineralogy, and zoology, including the study of venomous animals. C. Phisalix began investigating salamander venom and later collaborated with Gabriel Bertrand on viper venoms. Between 1889 and 1891, C. Phisalix published five articles in which he described the neurotoxic, respiratory, and thermal effects of salamander poison on experimental animals, as well as the possibility of inducing immunity through successive injections of low doses of the poison. G. Bertrand (1867–1962), a French chemist and biologist, entered the MNHN in 1886. He initially served as an assistant in a laboratory of plant physiology applied to agriculture (1889–1890), and subsequently in a laboratory of chemistry applied to organic substances (1890). A strong friendship developed between C. Phisalix and G. Bertrand (Figure 1). G. Bertrand later joined the Institut Pasteur in 1900, where he developed biological chemistry. Encouraged by A. Chauveau, C. Phisalix began studying venoms with the idea that venoms are possibly similar to bacterial toxins. He hypothesized that the methods of toxigenic bacteria attenuation for vaccination purposes could be applied to venoms. Indeed, following the success of L. Pasteur in preventing fowl cholera (1880), anthrax in sheep (1881), and rabies (1885) with the attenuated bacteria or viruses responsible for these diseases, C. Phisalix discovered a way to inactivate venom by heating it at 80 °C for 5 min and subsequently using it to vaccinate guinea pigs. In 1894, C. Phisalix and G. Bertrand presented their work at the Society of Biology in Paris on the immunization of guinea pigs with heat-attenuated venoms, and the subsequent treatment of venom-intoxicated animals with the serum of immunized guinea pigs. This marked the beginning of antivenomous serotherapy [4,5]. Moreover, C. Phisalix and G. Bertrand discovered that, unlike salamanders, vipers contain substances in their blood that confer resistance to their own venom. They also identified a natural resistance to viper venom in two species of grass snakes. Venom inhibitors in the blood of numerous snakes and mammals were later characterized (see below).

**Figure 2:**
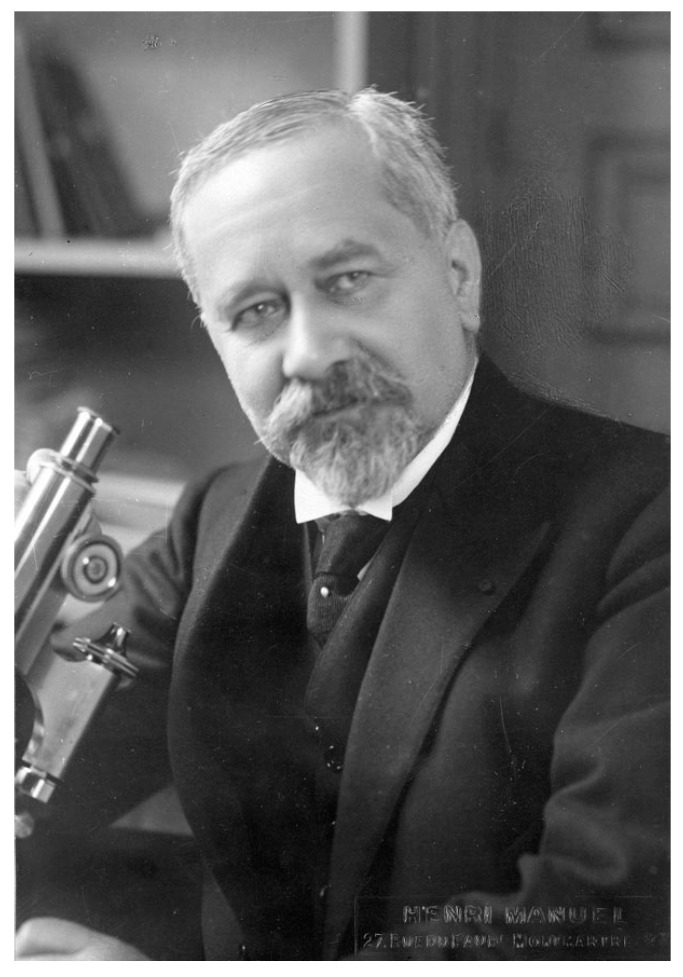
Albert Calmette (1863–1933) in his laboratory around 1920–1925. Photo was taken by Henri Manuel, Institut Pasteur/Musée Pasteur.

Albert Calmette (1863–1933) (Figure 2), a French military physician, followed the course of “microbie technique” directed by Emile Roux at the Institut Pasteur, Paris, in 1890. The next year, E. Roux sent A. Calmette in Saigon to establish the first Institut Pasteur network site, initially dedicated to the preparation of anti-rabies and anti-smallpox vaccines. During his stay in Indochina, he had the opportunity to investigate snake venoms. Back to France in 1893, he was sent to Lille to install a second Institut Pasteur (Institut Pasteur Lille), which he directed from 1895 to 1919. He tried to develop an immunization against cobra venom by adapting techniques used for bacterial toxins. However, cobra venom is resistant to heating. He then tried inoculating escalating doses, starting from sublethal doses, as well as alternative ways to inactivate snake venom by using various chemical treatments. A. Calmette succeeded in obtaining complete immunization using cobra venom treated with sodium hypochlorite, and he also demonstrated that sera from immunized animals have preventive and therapeutic effects against venom intoxication. He presented his results on antivenomous therapy in 1894 at the same session of the Society of Biology as C. Phisalix and G. Bertrand. A rivalry between the two scientist groups emerged for several years. The French National Academy of Sciences awarded the Monthyon prize to C. Phisalix and G. Bertrand for the discovery of an antivenom serum in 1894. However, A. Calmette claimed that his protocol for venom attenuation was more efficient than that of C. Phisalix. Indeed, snake venoms are more or less thermostable, and the chemical attenuation was more effective. Unfortunately, C. Phisalix died prematurely at age 54, and A. Calmette emphasized the importance of the antivenom serotherapy and his results at the international level. A. Calmette published numerous articles in French, English, and German, and he presented the interest and efficiency of the antivenom serotherapy at the Royal College of Physicians and Surgeons of London in 1896. It was found later that Calmette’s protocol of immunization induced highly neutralizing specific IgG antibodies, whereas those of C. Phisalix and G. Bertrand triggered mainly IgM. A. Calmette thought that the sera obtained after immunization with one snake venom could protect against the venom from different snake species. He produced antisera against cobra venom on a large scale at the Institut Pasteur of Lille for therapeutic use in humans. Then, the production of antivenom sera was undertaken in various countries: Brazil (Butantan Institute, 1901), the USA (Philadelphia, 1902), Australia (Sydney, 1902), India (Hafkine Institute of Mumbai, 1903; Kasauli, 1907), South Africa (Johannesburg, 1903), and the UK (London, 1905) [4,5,6,7,8,9].

## 3. Period of 1923–1978: Development of Antivenom Sera and Initial Characterization of Venom Constituents

**Figure 3:**
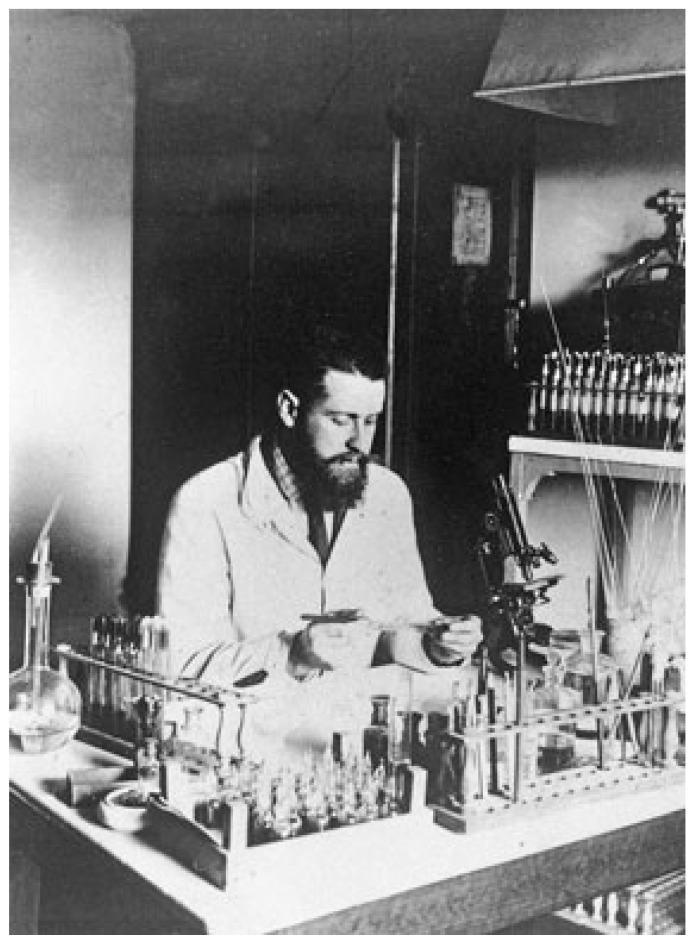
Gaston Ramon (1886–1933) in his laboratory around 1925. Photo was provided by the Institut Pasteur/Musée Pasteur.

**Figure 4:**
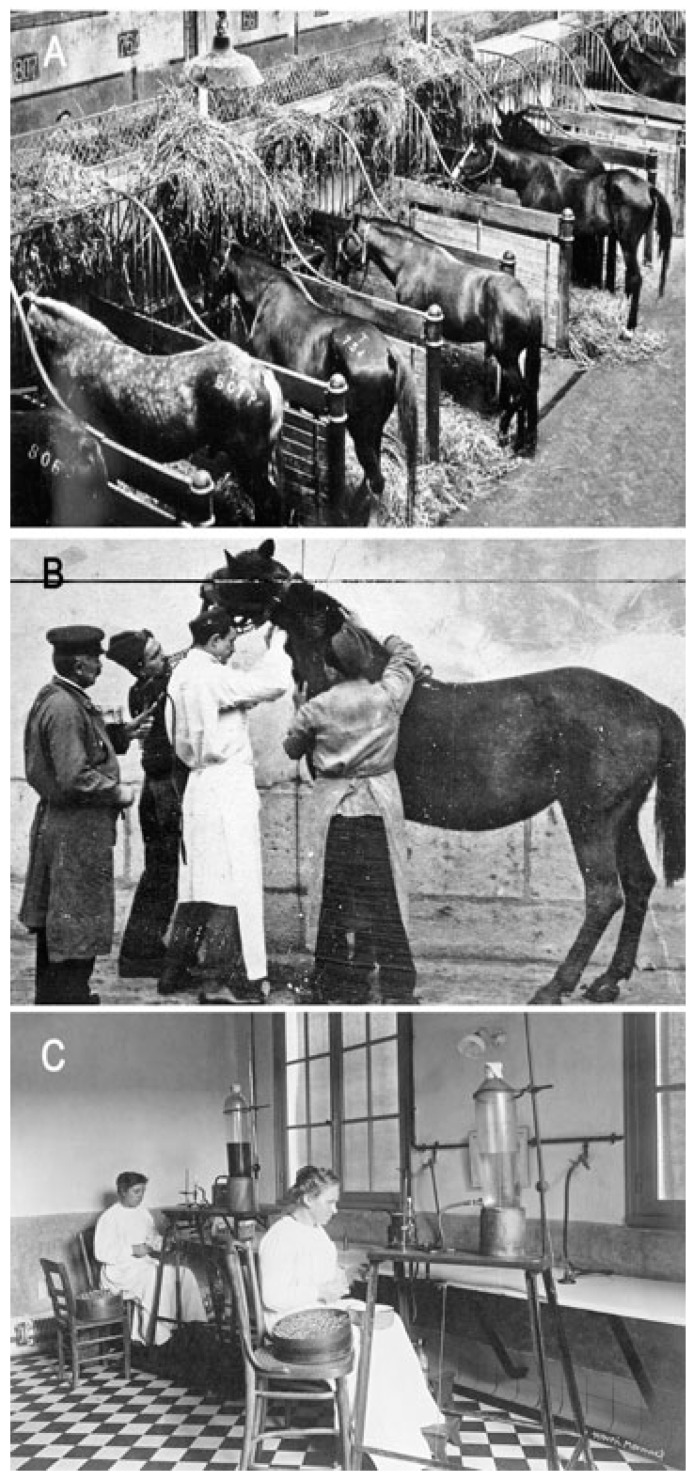
Institut Pasteur of Garches, production of horse antisera. (**A**) Horse stables; photo was provided by the Institut Pasteur/Musée Pasteur. (**B**) Serum sampling in a horse; photo was provided by the Institut Pasteur/Musée Pasteur. (**C**) Serum bottling; photo was taken by Henri Manuel, Institut Pasteur/Musée Pasteur.

Gaston Ramon (1886–1963) (Figure 3), a French veterinarian and biologist, was recruited by E. Roux to produce horse antisera at the Institut Pasteur Garches Annex. The property of Garches, in the Paris suburb of Marnes la Coquette, was gifted to L. Pasteur by the government in 1884 for his studies on rabies. This property, which was part of an old castle (castle of Villeneuve l’Etang), sufficiently distant from residential areas, was more favorable for maintaining a kennel of rabid dogs than Pasteur’s laboratory at the Ecole Normale Supérieure in downtown Paris. The Institut Pasteur in Paris was founded through a public international subscription and was inaugurated in 1888. It was dedicated to the treatment of rabies, basic research into infectious diseases, and training on microorganisms (with the first “microbie technique” course taught by E. Roux in 1888). After Pasteur’s death in 1895, the Garches Annex was used for the production of antisera in horses and investigations into animal immunization (Figure 4). Louis Martin (1864–1946), a French physician and biologist, was the assistant of E. Roux (1893–1894) and contributed to the development of anti-diphtheria serotherapy. L. Martin was appointed as the deputy head of the laboratory responsible for the production of an anti-diphtheria toxin and, subsequently, an anti-tetanus toxin (1894–1909).

G. Ramon was assigned to the serum production laboratory at the Garches Annex by E. Roux from 1911 to 1920 under the supervision of André-Romain Prévot [10]. In 1923, G. Ramon developed an innovative method of toxin inactivation based on a treatment with formalin. Thus, he demonstrated that the diphtheria toxin treated with a low dose of formalin became inactive, yet was able to induce a potent immunological response. He termed this novel form of toxin an “anatoxin” [3]. The idea to use formalin came from the fact that he applied this compound as an antiseptic for the preservation of therapeutic sera. He also used formalin for preserving standardized toxin samples, and he noticed that toxins treated with formalin were harmless, stable, and immunogenic [11]. This inactivation process was applied to other bacterial toxins, such as the tetanus toxin, as well as to venom. Indeed, in 1924, G. Ramon showed that snake venom could be inactivated by formalin to create a product that he referred as an “anavenom”. This method, which proved more reliable than a heat treatment or other chemical inactivation methods, was used for the immunization of horses and the production of antivenomous sera. Furthermore, G. Ramon introduced the concept of immunity adjuvants. He showed that the induction of local inflammation with calcium chloride or alum (aluminum hydroxide) could enhance the immune response to anatoxins. From 1926 to 1944, G. Ramon was the director of the Garches Annex, where he coordinated the production of anatoxins and antisera [12,13].

Paul Boquet, a French physician and scientist, joined the Institut Pasteur Garches Annex in 1933. He directed a laboratory specializing in antivenomous serotherapy, which was renamed the Laboratory of Venoms and Antivenomous Sera, and he held this position until 1978. P. Boquet conducted extensive research on the composition of snake venoms, notably from various species of Vipers and Naja. He developed novel in vivo and in vitro methods to discern the different toxic properties and enzymatic activities of venoms. The separation of venom constituents was achieved through electrophoresis and liquid chromatography. P. Boquet demonstrated through immunoelectrophoresis that certain toxic constituents are present in the venoms of multiple snake species, facilitating the cross-neutralization of snake venoms with antivenomous sera from different snake species. A key objective was to characterize the unique properties of venom constituents and to select the most effective antigens for creating polyvalent sera through horse immunizations. He proposed the classification of snake venoms into three groups based on their molecular size and serological properties. P. Boquet characterized the major lethal factor of the *Naja* snake’s venom, which is a small basic peptide of 61 amino acids called an alpha toxin, and he showed that it is produced by various *Elapidae* snakes. In collaboration with France Tazieff-Depierre, P. Boquet discovered that this factor exhibits activity similar to that of curare. Furthermore, the tritiated alpha toxin from the *Naja* snake (prepared by André Menez, CEA, France) was used to isolate the cholinergic receptor of the electric organ of *Electrophorus* by Jean Pierre Changeux, Institut Pasteur. P. Boquet also investigated the coagulation activity of venoms and phospholipases (PLAs), hypothesizing that PLAs might facilitate the entry of toxins into the nervous system. Moreover, P. Boquet supervised the production and control of antivenomous sera, which the Institut Pasteur distributed to numerous countries, notably Africa and Asia. In 1966, P. Boquet reported that 143 horses were used to produce 4345 L of antivenomous sera, leading to the preparation of 246,176 therapeutic doses of 10 mL each. He also contributed to the development and validation of international methods of antivenomous serum titration as well as the preparation of reference standards of venoms and sera in cooperation with the World Health Organization (WHO) [14,15,16,17,18,19,20,21,22,23,24,25,26,27].

France Tazieff-Depierre (1914–2006), a French pharmacist, entered the Institut Pasteur in 1934 in the Unit of Therapeutic Chemistry and then in the Unit of Protein Chemistry until 1979. Initially, she was interested in curararizing compounds and in the role of Ca^++^ in neurotransmitter release at the neuromuscular junction. She identified several classes of curararizing compounds, including those preventing acetylcholine activity and those with a depolarizing activity. These studies were completed by an investigation into the antagonists of curararizing compounds, such as acetylcholinesterases. F. Tazieff-Depierre showed that Ca^++^ activates acetylcholinesterases in vitro, but prevents their activity when fixed to the acetylcholine receptor. Starting in 1966, she was involved in animal toxins. P. Boquet asked her to analyze the mechanism of the paralytic effects of cobra venom. F. Tazieff-Depierre showed that the alpha toxin from *Naja nigricollis* binds with a high affinity to the acetylcholine receptor and that the paralytic effects are antagonized by anti-acetylcholinesterases, which increase the acetylcholine levels. In contrast, the gamma toxin from *Naja nigricollis* is cardiotoxic and induces muscle paralysis by the excessive Ca^++^ release from muscle fibers. The toxins from scorpion venom share a similar activity on striated muscle. Moreover, toxins from scorpions and sea anemones enhance the release of acetylcholine from neuronal endings through an increased intra-terminal Na^+^ concentration and the possible subsequent Ca^++^ release from internal stores, which triggers neurotransmitter release. Interestingly, due to their effect on acetylcholine release, these toxins are able to restore the neuromuscular transmission inhibited by botulinum toxin A. The numerous and pertinent works of F. Tazieff-Depierre shed light on the mode of action of some venom toxins, and also on the molecular mechanism of neurotransmission, notably on the role of Ca^++^ [28,29,30,31,32,33,34,35,36,37,38,39,40].

Several other scientists at the Institut Pasteur were also involved in venom research during this period. Camille Delezenne (1868–1932), a French physician, was recruited by Emile Duclaux to manage a physiology laboratory at the Institut Pasteur in 1900. He showed that the hemolytic and coagulase properties of certain snake venoms resulted from specific zinc-dependent enzymatic activities [41]. Marcel Rouvier (1898–1981) joined the Institut Pasteur in 1940 and successively directed the laboratories of the tetanus toxin, the diphtheria toxin, and the “Service des Anaérobies” from 1956 to 1968. Among his works on toxins, he studied the antigenicity of certain snake venoms, such as those from *Vipera aspis.* He also investigated various methods of preserving antivenomous sera for optimal therapeutic use, despite drastic field constraints such as exposure to heat [42].

## 4. Period of 1972–2004: Optimization of Serotherapy, Characterization of Biological Activity of Venom Components, and Natural Inhibitors from Snake Blood

**Figure 5:**
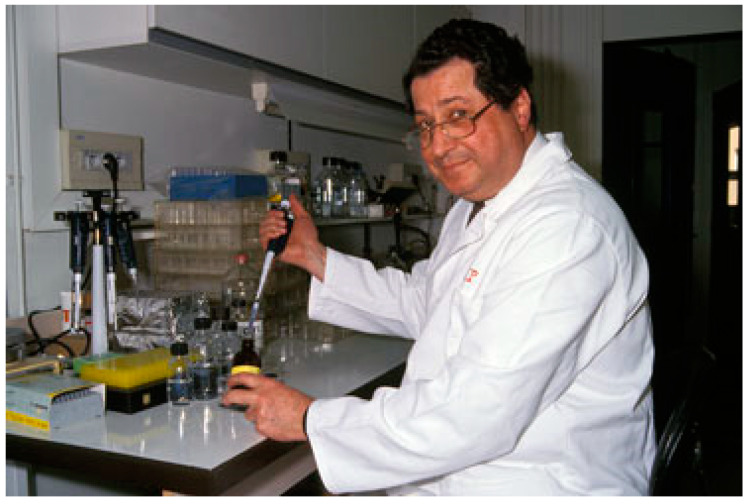
Cassian Bon (1944–2008) in his laboratory in 1995. Photo was provided by the Institut Pasteur.

Cassian Bon (1944–2008) (Figure 5) was born in Vietnam and completed his studies at the Ecole Normale Supérieure in Paris. In 1972, he joined the Institut Pasteur in Paris and worked on scorpion venoms with F. Tazieff-Depierre. This experience proved decisive, setting the course of his career on the study of venoms, primarily snake venoms, and antivenom sera across his dual scientific roles at the CNRS (Centre National de la Recherche Scientifique) and the Institut Pasteur [43]. C. Bon defended his PhD in 1979 on the major neurotoxins in snake venoms, ceruleotoxin and crototoxin. He later became a research scientist at the CNRS and led a venom laboratory at the Institut Pasteur. This laboratory was part of the cellular pharmacology unit directed by Bernardo-Boris Vargaftig (1937-), a Brazilian-born scientist who completed his medical studies in Sao Paulo (1963) and his scientific studies in Paris (PhD 1972). The research unit of B. Vargaftig evolved into the unit of “Pharmacology of the Mediators of Inflammation and Thrombosis” (1985–1997), and then the unit of “Biology, Cellular and Molecular Pharmacology of Pulmonary Inflammation” (1998–2004). B. Vargaftig is well known for his research on the role of the platelet-activating factor, particularly in bronchoconstriction and platelet aggregation.

In 1990, C. Bon was appointed as the director of the “Unité des Venins” at the Institut Pasteur, a role he held until 2004 [43]. His research focused on the mechanism of the presynaptic neurotoxicity of *Viperidae* toxins in snake venom, and he later developed studies on antivenom immunotherapy, both in experimental and human cases. This line of inquiry followed the Pasteur tradition initiated by Albert Calmette in 1894. C. Bon, along with his collaborators, studied the functional chaperon role and synergistic action of the acidic subunit of crotoxin. Crotoxin is a heterodimeric β-neurotoxin from the venom of the South American rattlesnake *Crotalus durissus terrificus.* The venom was purchased from the Instituto Butantan (Sao-Paulo, SP, Brazil) and crotoxin was purified from the venom using low-pressure gel filtration and ion-exchange chromatography. It is a toxic protein formed by the non-covalent association of a basic PLA_2_ subunit of low toxicity (CB) and a nontoxic acidic subunit (CA) devoid of catalytic activity that potentiates the toxic effect of CB [44]. Crotoxin binds to the presynaptic membrane, inducing the complete failure of neuromuscular transmission by impairing the release of acetylcholine at the neuromuscular junctions [45]. The CA subunit enhances the capacity of CB to reach its target at the neuromuscular junction, thereby increasing its lethal potency, but it reduces the enzymatic activity of CB [46,47,48,49,50].

Grazyna Faure, a Pasteurian collaborator of C. Bon, discovered several isoforms of crotoxin in individual venom samples of *Crotalus durissus terrificus* [51]. She purified 16 natural isoforms of crotoxin and compared their molecular structures and biological activities [52]. The work of G. Faure and C. Bon demonstrated that crotoxin variants result from the random association of four isoforms of the CA subunit (CA_1–4_) and four isoforms of the CB subunit (CBa_2_, CBb, CBc, and CBd). They described two classes of crotoxin, Class I and Class II, which differ in their pharmacological properties [52]. The comparison of crotoxin isoforms revealed that the stability of the complex plays a major role in its pharmacological action [53]. The origins of the isoforms were also identified. Multiple CA isoforms result from post-translational modifications occurring on a precursor pro-CA, while CB isoforms result from the expression of different messenger RNAs [54,55,56]. The presence of various PLA_2_ isoforms with diverse pharmacological activities could be explained by the accelerated evolution of exon regions after the duplication of a common ancestral gene, indicating the rapid adaptation of snakes for defense and predation.

During work in the Unité des Venins, G. Faure and Igor Krizaj demonstrated that crotoxin binds with a high affinity to a protein receptor from the presynaptic membrane of neuromuscular junctions in the electric organ of *Torpedo marmorata* [57,58]. They purified a 48 kDa crotoxin acceptor protein from *Torpedo* (CAPT) and characterized its binding to the receptor by surface plasmon resonance (SPR) characterization [58,59]. This study showed the formation of a ternary complex, CA CB Receptor, and the dissociation of CA at equilibrium.

Furthermore, G. Faure and Jonas Perales in the Unité des Venins identified, purified, and characterized a natural PLA_2_ inhibitor, the crotoxin inhibitor from *Crotalus* serum (CICS), from the blood of *Crotalus durissus terrificus* [60,61,62]. The CICS is an acidic 130 kDa oligomeric glycoprotein formed by the non-covalent association of 23–25 kDa subunits. This natural PLA_2_ inhibitor protects the rattlesnake from its own venom. It neutralizes the lethal and PLA activities of crotoxin, CB, and other PLA_2_s from the *Viperidae* family by binding to the CB subunit and preventing the association of CB with CA. Since the molecular mechanism underlying the interaction between crotoxin and CICS seems to be identical to that of crotoxin with its protein receptor CAPT, it was suggested that CICS acts physiologically as a false soluble crotoxin receptor, retaining the toxin in the vascular system of the snake and thereby preventing its toxic effects on the neuromuscular junction [60].

Valerie Choumet and colleagues from the C. Bon unit investigated the immunological aspects of crotoxin and other neurotoxic PLA_2_s [63,64,65,66,67,68,69]. Notably, they discovered that the acidic subunit CA of crotoxin interacts with the basic, single-chain ammodytoxin or agkistrodotoxin from *Viperidae* venom, in agreement with the amino acid sequence similarities between CB and these single-chain toxins with PLA_2_ activity. They also produced monoclonal antibodies (mAbs) against the crotoxin CA and CB subunits and determined their dissociation constants, cross-reactivity, and neutralization ability. This research led them to propose functional regions in the crotoxin components, the toxic and enzymatic sites on CB, and to suggest interacting regions on the two components. Further crystallographic studies by G. Faure’s group established the CA-CB binding interface of crotoxin [70]. The knowledge of this interface could be useful in mapping the epitope of the neutralizing monoclonal antibody A56.36.

Subsequently, C. Bon and his colleagues at the Institut Pasteur explored the envenomation process and its treatment by serotherapy. They determined the toxicokinetics and toxicodynamics of snake and scorpion venom in experimental animal models such as rats and rabbits. The distribution of venom in the organism was analyzed after an injection by the intravenous or intramuscular route and in the absence or presence of mAb fragments, either able (Fab) or unable (Fab’2) to cross the renal route. Toxin levels were assessed using an enzyme-linked immunosorbent assay (ELISA) and radiolabeled proteins. The kinetic parameters were analyzed after the administration of varying toxin doses. After an intravenous injection, the toxin exhibited a biexponential decline corresponding to the distribution and subsequent elimination. An intramuscular injection allowed the toxin to reach the vascular compartment, after which it exhibited a monoexponential decrease. The severity of symptoms was correlated with the toxin levels in the plasma. An intravenous injection of antivenom serum was the most efficient route. Antivenom antibodies were able to neutralize the totality of the toxin in the vascular compartment. Fab fragments were less efficient than Fab’2 fragments, possibly due to their differing pharmacokinetics. It is likely that complexes of antibody fragments/venom constituents were eliminated by phagocytosis rather than the renal route due to their high molecular weight. Interestingly, treatment with Fab was found to induce oliguria, which might induce adverse effects of serotherapy in humans. When examining experimental scorpion envenomation in rats, it was found that a Fab’2 intravenous injection neutralized venom more rapidly than Fab. However, Fab was more effective when administrated intramuscularly. Furthermore, Fab was more efficient at preventing the early symptoms of envenomation than Fab’2. Both antibody fragments were equally effective at preventing late symptoms, irrespective of the administration route. Based on these results, it was recommended to intravenously inject a combination of Fab and Fab’2 for the treatment of scorpion envenomation [66,71,72,73,74,75,76,77,78,79,80]. This research allowed for the optimization of the envenomation treatment by serotherapy. C. Bon was a renowned international expert in this field and was regularly invited by the WHO to share his expertise on this topic.

C. Bon and his collaborators began exploring the involvement of snake venoms in blood coagulation, aiming to develop novel therapeutic molecules for treating thrombotic events. Phospholipids (PLs) play an important role in the coagulation process. Anionic PLs are exposed on the membrane of activated platelets and facilitate the formation of complexes containing PL and protein enzymes involved in the coagulation cascade. Certain mammalian-secreted PLA2s and some snake PLA2s exhibit an anticoagulant effect through PL hydrolysis and/or by competing with coagulation factors, thus preventing their assembly into complexes involved in the coagulation cascade. Anticoagulant PLA2s have been reported in venoms from *Viperidae*, *Crotalidae*, *Elapidae*, and *Hydrophidae*. An analysis of snake PLA_2_ has helped to better define the mode of action of the anticoagulant PLA_2_s. Conversely, snake venoms also contain metalloproteases that activate coagulation factors, inducing procoagulant activity. C. Bon and collaborators purified and characterized some of these proteases, such as the prothrombin activator from *Bothrops atrox*, a serine protease from *Bungarus fasciatus* that activates blood coagulation factor X, and a thrombin-like serine protease from *Bothrops lanceolatus* that activates fibrinogen into fibrin [81,82,83,84,85,86,87,88,89,90,91,92].

C. Bon was actively involved in teaching about venomous animals at the MNHN and about human envenomation and its treatment at various universities. He was active in the international network of the Institut Pasteur, which produced antivenomous sera, notably in Algeria, Tunisia, Morocco, and Iran. He was a member of numerous scientific societies, including the French Society of Biochemistry and Molecular Biology, the French Association of Pharmacologists, the Society of Neurosciences, the International Society of Toxinology, and the International Society of Thrombosis and Hemostasis. He also served on the scientific council of the Institut Pasteur of Paris (1995–1999) and the Institut Pasteur of Iran (1998–2004). C. Bon was a co-founder of the French Society for the Study of Toxins (SFET, Société Française d’Étude des Toxines) in 1992 and served as president from 2000 to 2008. In 2004, the unit of venoms at the Institut Pasteur was closed, and C. Bon moved to the Laboratory of Chemistry of Natural Substances of MNHN. He was warmly welcomed by Max Goyffon, finding a familiar environment for teaching. During his last years, he was primarily focused on teaching, advising the WHO on envenomation, and organizing SFET meetings [43,93].

## 5. Period of 2004–2023: Novel Therapeutic Peptides Based on Snake Toxins and Structural Analysis of Their Binding Interface with Biological Targets

G. Faure, an expert research associate at the Institut Pasteur, continued the work on the structure–function relationships and the mechanisms of action of snake venom toxins with various protein targets, including the coagulation factor Xa, proton-gated ion channels, and the cystic fibrosis transmembrane regulator (CFTR) chloride channel. She led a group on PLA_2_ toxins in the Structural Immunology Unit (2004–2011) directed by Graham Bentley, and later in the channel-receptors unit (2011–2023) directed by Pierre-Jean Corringer.

Collaborating with Frederick Saul in the Structural Immunology Unit, Faure’s group determined the crystal structure of the heterodimeric crotoxin from *Crotalus durissus terrificus*, isoform CA_2_CBb of Class I [70], two isoforms of the basic CB subunit of crotoxin (CBb and CBd) [70,94], and two isoforms of ammodytoxin (AtxA and AtxC) from *Vipera ammodytes ammodytes* [95]. The three-dimensional structure of crotoxin revealed the nature of the binding interface between the CA and CB subunits and allowed for the identification of the key amino acid residues responsible for significant differences in the stability, toxicity, and enzymatic activity of the two classes of crotoxin complexes [70,96].

Snake venom PLA_2_s exhibit a wide range of toxic and pharmacological effects, including neurotoxic (pre-synaptic or post-synaptic), myotoxic, and cardiotoxic activities; anticoagulant effects; the inhibition of platelet aggregation; hemolytic activity; internal hemorrhage; anti-hemorrhage activity; convulsing and hypotensive activity; the induction of edema; organ necrosis or tissue damage; bactericidal, anti-humoral, anti-HIV (human immunodeficiency virus), anti-*Leishmania*, and anti-*Plasmodium* activity; and anti-viral activity against the dengue and yellow fever viruses [97]. Faure’s group identified a number of *Viperidae* venom PLA_2_s that inhibit blood coagulation factor Xa (FXa) via a non-catalytic PL-independent mechanism [98]. The interaction sites on PLA_2_ and FXa were mapped using SPR protein–protein interaction measurements, mutagenesis studies, and molecular docking simulations [98,99]. Comparative structural studies of natural PLA_2_ isoforms, which differ in their neurotoxicity and anticoagulant activity, contributed to a better understanding of their mode of binding to human FXa and calmodulin [95,96,100]. Faure’s group also discovered that PLA_2_ binding to FXa prevents the oligomerization of PLA_2_ [101]. The identification of the anticoagulant sites on Atx and CB and an analysis of the spatial arrangement of the PLA_2_-FXa interface led to a better understanding of the hemostatic process. These results are important for the elaboration of novel anticoagulant agents (non-competitive FXa inhibitors) [100].

**Figure 6:**
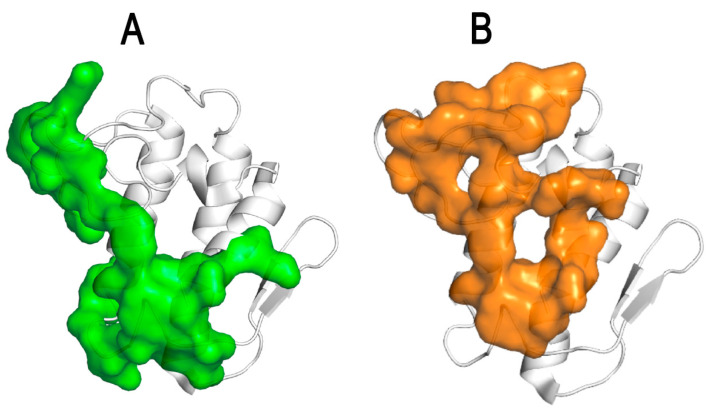
Identification of the pharmacological binding sites of CB, the PLA_2_ subunit of crotoxin, which is important for the structure-based design of new anticoagulant and anti-cystic fibrosis agents. (**A**) FXa-binding site of CB (in green) (adapted from Nemecz et al., 2020) [94]. (**B**) F508CFTR-binding site of CB (in orange) (adapted from Nemecz et al., 2020) [94].

Working in the channel-receptors unit at Pasteur, Faure’s group along with other collaborators discovered that the CB subunit of crotoxin binds with a high affinity to novel protein targets such as the pentameric proton-gated channel GLIC [102] as well as the cystic fibrosis transmembrane regulator (CFTR) and its mutant ΔF508-CFTR, which is implicated in cystic fibrosis [103]. They demonstrated that CB is a negative allosteric modulator of GLIC [102] and a positive allosteric modulator of CFTR [103]. By a direct interaction with the nucleotide-binding domain NBD1 of CFTR, CB potentiates the chloride channel current and corrects the trafficking defect of misfolded ΔF508CFTR inside the cell [103]. Faure’s group identified the CB-ΔF508CFTR interface by molecular docking and by HDX-MS (hydrogen–deuterium exchange–mass spectrometry) studies [103]. For the therapeutic development of new anti-cystic fibrosis agents, G. Faure and collaborators used a structure-based in silico approach and designed peptides mimicking this CBb-ΔF508NBD1 interface [104]. Using electrophysiological and biophysical methods, they identified several peptides that interact with the ΔF508NBD1 domain of CFTR and increase the chloride channel activity [104]. These significant results provide a new class of CFTR potentiators and describe a novel approach for developing therapeutic peptides for the treatment of cystic fibrosis. Thus, the biochemical and structural characterization of the functional and pharmacological sites of snake venom PLA_2_ (Figure 6) or fragments of PLA_2_ in complexes with their biological targets is essential for the structure-based design of novel therapeutic agents [94].

## 6. Concluding Remarks

Venomous animals pose a significant threat, and it has long been a concern to develop effective measures for the prevention and treatment of envenomation. Following in the tradition of the emerging field of microbiology during Pasteur’s era, C. Phisalix and G. Bertrand from the MNHN and A. Calmette from the Institut Pasteur achieved a breakthrough in combating envenomation. They demonstrated that prevention can be acquired by immunization with attenuated venoms and that treatment can be implemented through serotherapy. Later, G. Ramon refined the process of venom attenuation using formalin treatment. These discoveries led to the mass production and distribution of antivenomous sera, particularly to high-risk areas such as Asia and Africa, largely through the international network of the Institut Pasteur. In addition to applications for human health, an interest in the basic knowledge of venoms emerged, including investigations into venom constituents and their biological activities. Pasteurians have contributed widely to venom knowledge, as have scientists from national and international institutions outside the scope of this review (Supplementary Table S1). Briefly, at the French level, one should mention the contributions of the team led by André Menez (CEA) and later by Denis Servent on the structure/function relationships of snake and scorpion venom toxins, and that of Jordi Molgo and Evelyne Benoit (CNRS, CEA) on marine toxins and, notably, on conotoxins [105,106,107,108], as well as numerous colleagues from the Universities of Aix-Marseille (Pierre Bougis, Marie-France Martin-Eauclaire, Pascale Marchot, Hervé Rochat, …), Anger (Christian Legros, Cesare Mattei …), Côte d’Azur (Sylvie Diochot, Pierre Escoubas, Michel Lazdunski …), Nantes (Michel de Waard, Michel Ronjat …), or Montpellier (Sébastien Dutertre …). Regarding the French contribution, the annual SFET meetings, called “Rencontres in Toxinology”, offer an opportunity to present the most recent findings and updates in animal and bacterial toxins. The in-depth characterization of animal toxins allows for comparisons to be made with plant and bacterial toxins or, in contrast, allows for their unique properties to be highlighted. The extreme potency of toxins primarily results from their ability to target or hijack key physiological processes. Therefore, due to their high specificity for the host targets, toxins are very efficient tools for exploring cellular processes. The unique properties of certain toxins to reverse or inhibit specific pathological effects make them suitable therapeutic tools or enable the design of novel therapeutic approaches. Recent advances in animal toxins support their therapeutic application.

## Data Availability

Not applicable.

## Supplementary Materials

The following supporting information can be downloaded at: https://www.mdpi.com/article/10.3390/toxins15070462/s1, Table S1: Institut Pasteur’s collaborators working on animal toxins from 1986 to 2023.
