# Supplementary material for: Animal Toxins: A Historical Outlook at the Institut Pasteur of Paris
Source: Toxins (Basel). 2023 Jul 19;15(7):462. doi: 10.3390/toxins15070462 (PMC10467091; doi:10.3390/toxins15070462)
Supplement: Supplementary file 1 [file toxins-15-00462-s001.zip › toxins-2486681-supplementary.pdf]

Supplemental Table S1. Institut Pasteur's collaborators working on animal toxins from 1986 to 2023

Michel R. Popoff, Grazyna Faure, Sandra Legout and Daniel Ladant

Country, Institution, city	laboratory	Collaborators
France, Institut Pasteur, Paris	Laboratory of Venoms C. Bon in the Unit of Venoms directed by Boris Vargaftig 1986-1990<br><br>Unit of Venoms directed by C. Bon 1990-2004	Cassian Bon, Guy Marlas, François Radvanyi, Bernard Saliou, Annie Robbe-Vincent, Anne Wisner, Christiane Bouchier, Emmanuèle Delot, Carine Mounier, Valérie Choumet, Mireille Leduc, Sandrine Braud, Elisabeth Ferquel, Grazyna Faure, Kunihiro Ozutsumi, Elisabeth Myatt, Elisabeth Dumont, Sabine LePorrier, Jean-Marie Genevard, Muriel Chen-Kuo-Chang, Carine Vilette, Marlène Charnay, Petra Prijatelj, Igor Krizaj, Jonas Perales, Rachid C Maroun
	Unit of Structural Immunology directed by G. Bentley 2004-2011	Graham Bentley, Frederick Saul,<br>Grazyna Faure, Sylvain Wormser, Anne Fournier, Benoit Villette, Julie Renard, Haijin Xu, Mahdi Belaid
	Unit of Channels-receptors directed by P. J. Corringer 2012-2023	Pierre-Jean Corringer, Marie Prevot, Ludvic Sauguet,<br>Grazyna Faure, Maciej Ostrowski, Dorota Porowinska, Tomasz Prochnicki, Anne-Solenn Debris, Rafel Simo-Vicens, Norbert Odolczyk, Marc Ravatin
	Laboratory of Microsequencing, Unit of Molecular Biology of Channels	Luc Camoin, Jean-Luc Guillaume
	Hybridolab	Jean-Claude Mazié, Farida Nato, Pierre Lafaye
	Crystallography Platform	Frederick A. Saul, Ahmed Haouz
	Unit of Nuclear Magnetic Resonnance of Macromolecules and HDX-MS Technological Platform	J. Inaki Guijarro, Muriel Delepierre
	Platform of Molecular Biophysic	Patrick England, Sylvianne Hoos, Bruno Baron, Bertrand Raynal
France, Paris	Museum National d'Histoire Naturelle (MNHN)	Max Goyffon, Thérèse Montenay-Garastier, simone Puiseux-Dao, Christine Rollard, Jean-Philippe Chippaux, Philippe Billiald

France, Commissariat à l'Energie Atomique (CEA), Saclay	Département d'Ingénierie et d'Etudes des Protéines	André Ménez, Jean-Claude Boulain, Frederic Ducancel, Odile Tremeau, Pierre Fromageot, Denis Servent, Daniel Gillet
France, Sanofi, Vitry-sur-Seine	Intergated Drug Discovery, In vitro Biology	Jean-Marie Chambard
France, Centre National de Recherche Scientifique (CNRS), Gif-sur-Yvette	Laboratoire de NeuroBiologie Cellulaire et Moléculaire (NBCM)	Jordi Molgo, Evelyne Benoit
France, Faculté de Médecine Nord, Marseille	CNRS-Unité Mixte de Recherche (UMR) 6560 Protein Engineering	Hervé Rochat, Pascale Marchot, Pierre Bougis, Marie-France Martin-Eauclaire
France, Valbonne	Institut de Pharmacologie Moléculaire et Cellulaire	Michel Lazdunski, Gérard Lambeau
France, Faculty of Pharmacy, Univeristy of Paris-Sud Chatenay Malabry	Laboratory of Animal Biology	Françoise Goudey-Perriere
France, Centre de Recherches du Service de Santé des Armées (CRSSA), La Tronche	Unit of NeuroPharmacology	Guy Lallement, Frédéric Dorandeu
France, Institut Necker Enfants Malades, Paris	Institut National de la Santé et de la Recherche Médicale (INSERM) U1151	Aleksander Edelman, Isabelle Sermet-Gaudelus, Alexandre Hinzpeter, Nathalie Servel, Mario Ollero
France, Université Pierre et Marie Curie (UPMC), Paris	CNRS/UPMC Institut des Cordeliers	J. Teulon, S Lourdel
Poland, Nicolaus Copernicus University, Torun	Department of Biochemistry	Maria Stankiewicz, Maciej Ostrowski, Dorota Porowinska-Nemecz –
Poland, Polish Academy of Sciences, Warsaw	Institute of Biochemistry and Biophysics	Piotr Zielenkiewicz, Norbert Odolczyk, Michal Dadlez, Aiswarya Premchander
Germany, Frankfurt Universitat, Frankfurt	Zentrum des Rachstmedizin	Dietrich Mebs
Belgium, University of Leuven, Leuven	Laboratory of Toxicology	Jan Tytgat
Slovenia, Jozef Stefan Institute, Ljubijana	Department of Molceular and Biomedical Sciences	Igor Krizaj, Franc Gubensek, Vladka Curin-Serbec, Natasa Vucemilo, Alenka Copic, Jernej Sribar, Joze Pungarcar, Petra Prijatelj-Znidarsic

Russia, Russian Academy of Sciences, Moscow	Shemyakin-Ovchinnikov Institute of Bioorganic Chemistry	Victor I. Tsetlin, Yuri N. Utkin
Czech Republic, Faculty of Medicine, Praha	Institute of PathoPhysiology	F. Kornalik
Spain, Valencia	Instituto de Biomedicina, Consejo Superior de Investigaciones Cientificas (CSIC)	Juan Calvete
Scotland, University of Strathhclyde, Glasgow	Strathhclyde Institute for Drug Research, Department of Physiology and Pharmacology	Alan L. Harvey, E. G. Rowan
United Kingdom, Liverpool School of Tropical Medicine, Liverpool		Theakston R.D.G.
Canada, McGill University, Montréal	Departement of Physiology and Biochemistry	Gergely L. Lukacs
India, University of Mysore, Mysore	Department of Studies in Biochemistry	Veerabasappa T. Gowda
Singapore, National University of Singapore	Bioscience Centre, Faculty of Sciences	R. Manjunatha Kini, P. Gopalakrishnakone
Japan, Sophia University, Tokyo	Department of Chemistry	Nobuo Tamiya, Toru Tamiya
Brazil,Universidade Estadual de Campinas (UNICAMP), Campinas	Faculdade de Ciências Médicas (FCM)	Julia Prado-Franceschi, Albetiza Lobo de Araujo
Brazil, Instituto Oswaldo Cruz, Rio de Janeiro	Departamento de Fisiologia e Farmacodinamica	Jonas Perales, Christina Villela, Gilberto B Domont, Haity Moussatché
Brazil, Instituto Butantan, Sao Paulo	Laboratoy of Biochemistry and Biophysics	Ana Marisa Chudzinski-Tavassi
Brazil, Universidade Federal do Rio de Janeiro (UFRJ), Rio de Janeiro	Instituto de Bioquimica Medica, Centro de Ciencias da Saude	Russolina B. Zingali
Brazil, Universidade Federal de Minas Gerais, Belo	Departamento de Bioquímica e Imunologia	Maria Elena de Lima

Horizonte, Minas Gerais,		
Costa Rica, Universidad de Costa Rica, San José	Instituto Clodomiro Picado	José Maria Gutiérrez, Bruno Lomonte
Netherlands, University of Utrecht, Utrecht	Department of Enzymology and Protein Engineering, Center for Biomembranes and Lipid Enzymology	Hubertus M. Verheij
USA, The Scripps Research Institute, La Jolla, California	Department of Molecular and Experimental Medicine and Vascular Biology	John H. Griffin
USA, University of Washington, Seattle	Department of Chemistry and Biochemistry	Michael H. Gelb
USA, Arizona State University, Tempe	Department of Chemistry and Biochemistry	Allan L. Bieber
USA, Oklahoma State University, Stillwater	Department Anatomy, Pathology & Pharmacology	Charlotte L. Ownby
USA, University of Virginia School of Medicine, Charlottesville	Department of Microbiology	Jay W. Fox
Italy, University of Padova, Padova	Centro CNR Biomembrane and Dipartimento di Scienze Biomediche	Cesare Montecucco
Mexico, Universidad Nacional Autónoma de México, Maxico	Instituto de Biotechnologia, Departamento de Medicina Molecular y Bioprocesos	Lourival D. Possani
Algeria, Faculty of Biological Sciences, Algier	Laboratory of cellular and Molecular Biology	Laraba-Djebari Fatima, Djélila Hammoudi-Triki
Tunisia, Pasteur Institute of Tunis, Tunis	Laboratory of Venoms and Toxins	Mohamed El Ayeb, Naziha Marrakchi
China, the Chinese Academy of Sciences, Kunming, Yunnan	Kunming Institute of Zoology	Yulliang Xiong, Yun Zhang
Vietnam, National Poison Control Center, Hanoi	Venom Research & AV Production Unit	Trinh Xuan Kiem

Israel, Tel Aviv University, Tel Aviv	Department of Zoology	Elazar Kochva, Avner Bdolah
Switzerland, Atheris Laboratories, Geneva		Reto Stocklin
